# Retraining function in people with Parkinson’s disease using the Microsoft kinect: game design and pilot testing

**DOI:** 10.1186/1743-0003-11-60

**Published:** 2014-04-14

**Authors:** Brook Galna, Dan Jackson, Guy Schofield, Roisin McNaney, Mary Webster, Gillian Barry, Dadirayi Mhiripiri, Madeline Balaam, Patrick Olivier, Lynn Rochester

**Affiliations:** 1 Clinical Ageing Research Unit, Institute for Ageing and Health, Campus for Ageing and Vitality, Newcastle University, Newcastle upon Tyne NE4 5PL, UK; 2 Culture Lab, School of Computing Science, Newcastle University, Newcastle upon Tyne, UK

**Keywords:** Parkinson’s disease, Kinect, Rehabilitation, Balance, Exergaming

## Abstract

**Background:**

Computer based gaming systems, such as the Microsoft Kinect (Kinect), can facilitate complex task practice, enhance sensory feedback and action observation in novel, relevant and motivating modes of exercise which can be difficult to achieve with standard physiotherapy for people with Parkinson’s disease (PD). However, there is a current need for safe, feasible and effective exercise games that are appropriate for PD rehabilitation. The aims of this study were to i) develop a computer game to rehabilitate dynamic postural control for people with PD using the Kinect; and ii) pilot test the game’s safety and feasibility in a group of people with PD.

**Methods:**

A rehabilitation game aimed at training dynamic postural control was developed through an iterative process with input from a design workshop of people with PD. The game trains dynamic postural control through multi-directional reaching and stepping tasks, with increasing complexity across 12 levels of difficulty. Nine people with PD pilot tested the game for one session. Participant feedback to identify issues relating to safety and feasibility were collected using semi-structured interviews.

**Results:**

Participants reported that they felt safe whilst playing the game. In addition, there were no adverse events whilst playing. In general, the participants stated that they enjoyed the game and seven of the nine participants said they could imagine themselves using the game at home, especially if they felt it would improve their balance. The Flow State Scale indicated participants were immersed in the gameplay and enjoyed the experience. However, some participants reported that they found it difficult to discriminate between different types and orientations of visual objects in the game and some also had difficulty with the stepping tasks, especially when performed at the same time as the reaching tasks.

**Conclusion:**

Computer-based rehabilitation games using the Kinect are safe and feasible for people with PD although intervention trials are needed to test their safety, feasibility and efficacy in the home.

## Introduction

Parkinson’s disease (PD) is a multi-system neurodegenerative disorder that impairs postural control and mobility, impacting negatively on community ambulation [1] and increases the risk of slips, trips and falls [2]. Exercise is emerging as an effective therapy to improve gait, balance and mobility in PD [3,4]. Furthermore, it appears that the mode of delivery and content of exercise is important for the optimal long-term change in functional activities and this is thought to be due to more effective retraining of compensatory circuits within the brain [5]. Practice of complex tasks (total body movement rather than exercising a single joint), using sound and vision to enhance exercise, increased practice and knowledge of changes in performance have all been shown to be important features of exercise [5-13]. It is difficult to achieve this with standard physiotherapy and the intensity and opportunities to engage in PD specific exercise programs are limited due to access to physiotherapy services. Exercise-based computer games (exergames) such as those played with the Nintendo Wiitrade™, Sony Playstation Eye™ and Microsoft Kinect (Kinect) systems may help facilitate high volume and quality exercise to improve postural control and mobility in people with PD in the home. These systems may also be used as sensors to measure clinically relevant outcomes during gameplay [14].

Initial studies have produced promising results for the use of exergaming as a rehabilitation tool for older adults and people with neuropathies [15-23], including those with PD [24-29]. The current literature on exergaming for PD suggests that people with PD accept and enjoy playing exergames, are able to improve their gameplay with practice and that improvements in gameplay transfer to improvements in clinical measures of postural control [24-29]. However, there is little evidence in regard to its safety and its clinical effectiveness is yet to be established by large randomised clinical trials. In addition, all but one of these previous studies used commercially available games which are not specifically designed for PD. To our knowledge, there have been no exergames developed to improve postural control in people with PD. Therefore, we set out to design an exergame to improve the dynamic postural control of people with PD using the Kinect system.

The Kinect system is a camera-based controller which a player can use to directly control a game through body movement without the need for balance boards or handheld controllers. Another benefit of using the Kinect system is that its depth sensor allows measurement of three-dimensional movement patterns, which allows real-time feedback of movement whilst playing the game as well as home-based assessment of clinical outcomes and symptoms. Three dimensional reconstruction of body motion also permits the development of games that target specific coordination patterns when retraining movement, unlike other controller or force platform based exergaming systems. This feature may be useful to enhance the quality of training as well as help avoid injury or fatigue due to poor technique.

The specific aims of this project were to i) develop a simple game for retraining balance and postural control for PD, with input from people with PD and physiotherapists with expertise in PD; and ii) pilot test the prototype game with a group of people with PD to assess the game’s safety and feasibility.

### Part 1 - game design

Both computer games and exercise interventions need to be acceptable for the intended population [25,30]. Taking a user-centred design approach, we conducted a design workshop with people with PD to input into our game design to ensure the game was appropriate for people with PD.

## Methods

### Participants

Two people with mild to moderate (Hoehn & Yahr stage II and III) PD and one carer attended the design workshop. Parkinson’s disease support groups were contacted through a national PD charity, Parkinson’s UK. People with PD were included if they were between 40–80 years old and had mild to moderate PD. The design workshop consisted of one three-hour session. Participants took part in a discussion about the accessibility and usability of commercially available exergames (Nintendo Wii^TM^ and Microsoft Xbox Kinect) and exercise based computer games for people with PD. Participants were then provided the opportunity to play and view several types of games and comment on their appropriateness for people with PD. Finally, they were asked to discuss their thoughts on which features they would like included in a game targeting their postural control. We also asked participants about their daily use of technology and preferences for the style and type of game they would like us to develop. The session was video recorded and transcribed for inductive thematic qualitative analysis. Ethical approval was obtained from the Newcastle University Research Ethics Committee and all participants signed an informed consent form prior to this study.

## Results

### Design workshop

Several requirements relevant to game design emerged from comments made during the design workshop (Table 1).

**Table 1:** Participant comments during the design workshop that

**Concerns**	Did not like the idea of an adventure or complex narrative based game, especially science fiction
	Were concerned that the pace of the game should not be too fast
**Preferences**	Preferred the idea of solo play over play with others (self-conscious over performance)
	Seemed more attracted to ‘real life’ events than complex characterisation or fantasy elements
	Expressed a preference for cartoon style graphics over more realistic renderings
	Liked puzzles, although one participant expressed concern that combining puzzles with physical tasks might be overly complicated
	Expressed a preference for outdoor scenarios;
	Were able to identify with a cartoon avatar which mirrored their actions
	Enjoyed satisfying sound effects associated with actions (for example, the thwack of hitting a ball when playing a golfing game)

When observing our participants with PD play different types of games, we noted that one person had marked difficulty using the Nintendo Wii Fit™ balance board, which he had to repeatedly step on as part of a commercial dancing game. Two of the participants also found using the handheld Nintendo Wii™ controller frustrating when playing a golf game.

### Game design

**Figure 1:**
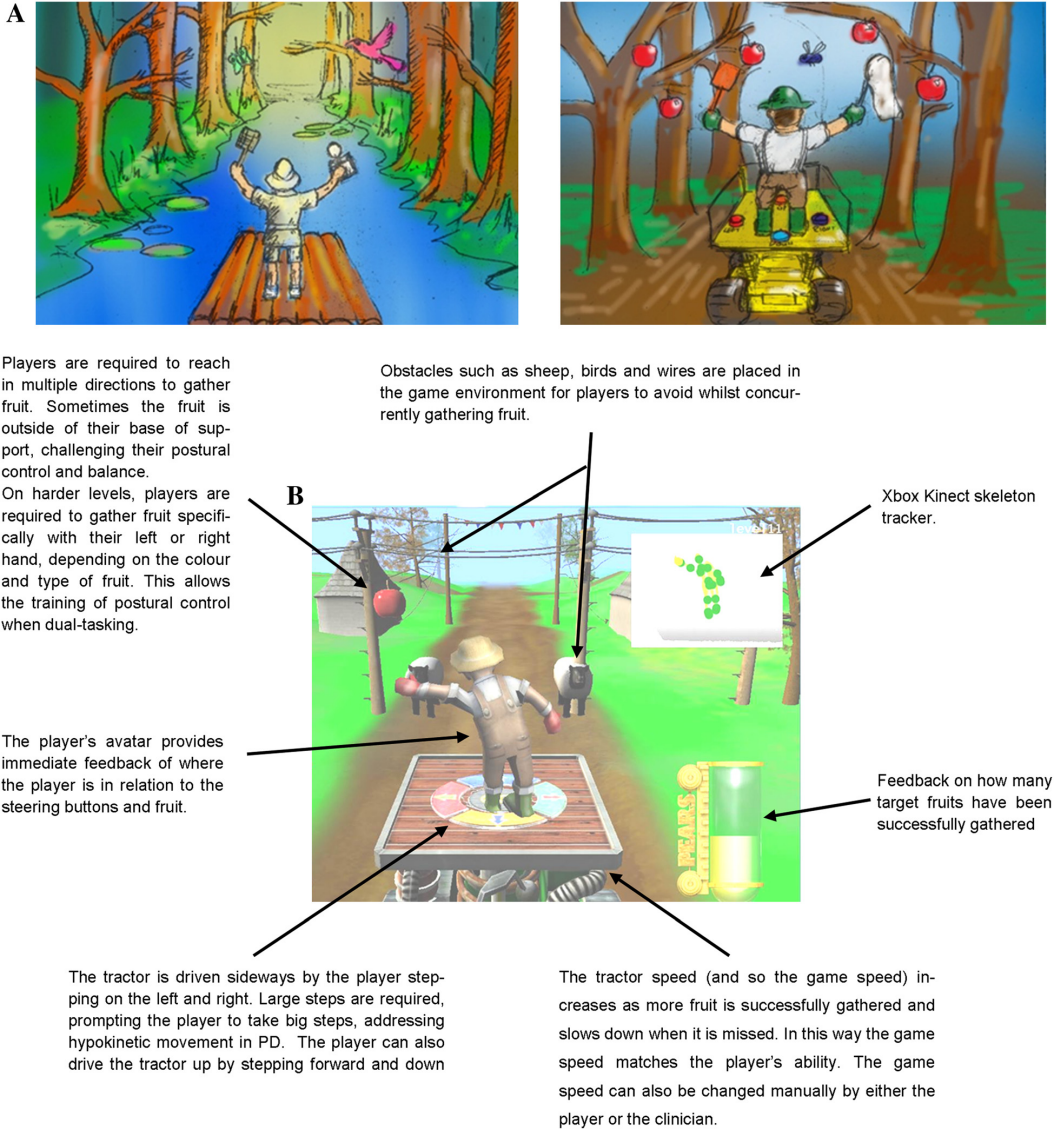
Conceptual game artwork based on the suggestions from the design workshop (Panel A) and an annotated screenshot of the final game highlighting important game features (Panel B).

Based on design requirements established through interactions with people with PD and an iterative design and development process between the research team’s physiotherapists, game designers and artists, a prototype game targeting postural control rehabilitation in people with PD was created. Microsoft’s research ‘Kinect for Windows SDK (software development kit)’ was used to provide an Application Programmer’s Interface to the Kinect sensor. The game was developed to train postural control by rewarding high volumes of reaching outside of the base of support and taking large multi-directional steps. Early sketches of game artwork and an annotated screenshot of gameplay are shown in Figure 1 (See Additional file 1: Video 1 for an example of gameplay). The premise of the game was that players took on the role of a farmer picking fruit from a tractor, a theme inspired by one of the design workshop participants. As the tractor moved through the environment, players had to reach out to pick fruit and drive (by stepping) to avoid obstacles.

To ensure people with PD did not initially find the game too complex, we structured the game with 12 levels of increasing complexity. Training of postural stability was informed by a theoretical model of balance dysfunction and focussed on three of the four domains: quiet stance, reactive postural adjustments and anticipatory postural adjustments [31]. The early levels of the game focussed solely on reaching (anticipatory adjustments), and introduced more cognitively challenging levels (reactive adjustments) whereby the hand with which the player picked the fruit was conditional on the type and colour of the fruit (i.e. “Pick the red apples with your left hand and the yellow pears with your right hand”). In doing so, the game promoted moving the centre of mass from quiet standing to outside the base of support. As the players progressed, they were required to drive the tractor to avoid oncoming obstacles such as sheep, high wires, birds and wasps. The tractor was driven by making large steps either forwards (tractor moves up), backwards (tractor moves down) and sideways. Promoting large steps was aimed at targeting hypokinetic movement demonstrated by people with PD, which is responsive to external cueing [32]. One foot had to stay in the centre of the tractor to ensure people took large steps instead of several small steps as well as to restrict the player’s movement within the capture volume of the Kinect sensor. In addition to hearing a positive noise for successfully collecting fruit (relatively high pitch), a bar at the side of the screen filled up to indicate better collection of the fruit throughout each level. At the end of each level, an encouraging noise (“crowd cheering”) played and the proportion of fruit successfully collected was displayed.

People with PD also often have difficulties performing motor tasks when dual-tasking [33,34]. Therefore, the final levels of the game were designed to train postural control under dual-task conditions, by requiring players to both pick fruit (reaching) whilst driving the tractor to avoid the obstacles (stepping) concurrently. The multi-directional stepping combined with reaching tasks further stimulated postural control under more dynamic conditions than just reaching alone. To ensure the game speed was appropriate the speed of the game could be manually adjusted by either the player or clinician and automatically slowed down if the player repeatedly missed fruit or collided with obstacles. Conversely, and the game became faster if the player successfully collected the fruit.

### Part 2 – safety and feasibility of gameplay

After the design phase, we sought to pilot test the game with a group of people with PD to assess its safety and feasibility, as well as obtain feedback about the gameplay. For the purposes of this article, we define safety as the ability to maintain postural control whilst playing the game, without slipping, tripping or falling. Feasibility was defined as the ability to play and improve gameplay performance, as well as the enjoyment and immersion in the gameplay.

## Methods

### Participants

Nine people with PD were recruited through local movement disorder clinics to play the game. Inclusion criteria: diagnosis of idiopathic PD (by a consultant neurologist with a specialist interest in movement disorders), absence of any other neurological problem or any severe co-morbidity likely to affect gait, absence of dementia, adequate sight and hearing with glasses or hearing aid if required, independently mobile indoors without a walking aid and no severe dyskinesias or prolonged off periods. Participants were tested at the peak dose of their anti-Parkinson’s medication.

### Demographic and clinical measures

Prior to gameplay, we documented participant age, sex, height and body mass. In addition, we measured motor disability using part III of the Movement Disorders Society version of the UPDRS (Unified Parkinson’s Disease Rating Scale) [35] and balance self-efficacy using the Activities-specific Balance Confidence scale [36].

### Setting and equipment

We wished to pilot test the game in a controlled laboratory setting to ensure its safety prior to testing its effectiveness in the home. To achieve this, participants attended the Movement Laboratory at the Clinical Ageing Research Unit, Newcastle University, to play the game. The game was displayed on a 1080x780 resolution LG plasma screen (1100 mm wide × 620 mm high), mounted 3 m away from the participant. We played the game through a laptop running Windows 7 to which the Kinect sensor was attached. The Kinect API’s skeletal tracking functions provided a position estimate for 20 anatomical measurements (including the head, shoulders, elbows, wrists, hands, hips, knees, ankles and feet) at a frequency of 30 Hz.

### Gameplay

Participants played the game for approximately 30 minutes. The game was presented with increasing levels of difficulty (Table 2). Participants were allowed to keep playing or to repeat levels if they wanted. We ceased level progression when participants were tired or if the levels were too complex for them to keep progressing. Unlike an intervention trial where the progression may be slower, allowing for more practice to master each level, the goal of this session was to expose participants to as much of the game’s content within the session, without pushing them to levels which either they or the researchers perceived as too cognitively or physically demanding. Each level lasted approximately 2 minutes, and participants were encouraged to comment and discuss the gameplay, highlighting things they liked or disliked. An experienced physiotherapist was present at each session to ensure the safety of the participant.

**Table 2:** Order and description of game levels

**Order**	**Reaching towards fruit**	**Stepping to avoid obstacles**
1	Either hand	-
2	Conditional: Left hand apples, right hand pears	-
3	-	Learning to “drive” the tractor up and down (stepping forwards and backwards) and sideways (stepping left and right).
4	-	Sideways
5^+^	Either hand	Sideways
6	Conditional: Left hand apples, right hand pears	Sideways
7	-	Forwards and backwards
8	Either hand	Forwards and backwards
9	Conditional: Left hand apples, right hand pears	Forwards and backwards
10	-	Sideways, forwards and backwards
11	Either hand	Sideways, forwards and backwards
12*	Conditional: Left hand apples, right hand pears	Sideways, forwards and backwards
5^+^	Conditional: Left hand apples, right hand pears	Sideways

*See attachment 1 for a video example of gameplay; ^+^Participants who progressed through all of the levels also played level 5 again to establish whether they improved with practice.

### Data extraction and synthesis

Gameplay data was recorded while participants played the game, including their body position which was recorded using the inbuilt Kinect skeletal model tracker. We also recorded the number of times participants reached for fruit or stepped to avoid obstacles as well as if these attempts were successful. All of the participants who progressed through all of the levels repeated Level 5 (stepping sideways and conditional reaching for fruit) to assess whether their gameplay improved with practice (i.e. successfully gathered more fruit per level). Data are described using means and ranges.

### Interview process

A semi-structured interview was conducted by the research physiotherapist after the gameplay. Questions focussed on perceptions of playing the game in terms of safety, gameplay, the game’s utility as an exercise intervention and the type of games participants already play at home. Interview questions are listed in Table 3. Participants were also asked to fill in a Flow State Scale questionnaire, which quantifies different perceptual domains of immersion during physical activity (See Table 4 for example items) [37].

**Table 3:** List of questions asked during the semi-structured interview

**Question number**	**Question**
**1**	**What type of games do you play at home?**
2	Who do you play with?
3	How often do you play and for how long?
4	Where in your home do you play?
**5**	**How often do you exercise?**
6	Do you exercise with anyone?
7	What types of exercise do you do?
8	Are you self-motivated to exercise?
9	Does anyone tell you to exercise? If so, who?
10	Would you consider the game you played an “exercise game”?
11	Did the game support the right kind of exercise for you?
**12**	**What did you think of the game you just played?**
13	Could you imagine playing it at home?
14	Would you want to play the game with anyone else?
15	Did you feel safe playing the game?
16	If the game were available to use at home, would you play it?
17	If the game were available to purchase, would you buy it?

**Table 4:** Individual and group response to the Flow State Questionnaire

**Construct**	**Example item**	**Participant**										
		**1**	**2**	**3**	**4**	**5**	**6**	**7**	**8**	**9**	**Mean**	**SD**
**Enjoyment (Autotelic experience)**	I really enjoyed the experience	5.00	3.25	2.25	4.25	4.75	4.75	3.50	4.25	4.25	4.03	0.88
**Clear goals**	My goals were clearly defined	5.00	5.00	2.75	4.50	5.00	5.00	3.25	4.00	3.50	4.22	0.88
**Challenge**	My abilities matched the high challenge of the situation	5.00	3.25	2.25	3.50	4.25	5.00	3.25	4.50	3.00	3.78	0.96
**Concentration**	My attention was focussed entirely on the game	5.00	4.50	3.50	4.75	5.00	5.00	4.50	4.75	4.00	4.56	0.51
**Control**	I felt in total control of my body, without conscious effort	5.00	3.50	2.00	3.00	4.25	4.25	2.25	3.75	3.00	3.44	0.98
**Feedback**	It was clear to me that I was doing well	5.00	4.50	2.25	3.50	4.00	4.75	4.00	4.00	3.00	3.89	0.87
**Action**	Things just seemed to happen automatically	5.00	2.75	2.00	2.50	3.50	4.50	2.50	1.75	3.50	3.11	1.10
**Transience**	Time seems to alter (either speed up or slow down)	5.00	1.00	3.25	2.50	2.00	2.00	2.50	2.25	3.50	2.67	1.14
**Loss of self-consciousness**	I was not worried about what others might be thinking about me	5.00	4.25	2.25	4.50	3.50	5.00	5.00	5.00	2.75	4.14	1.06

Cells represent the mean of four similar items and ranging from 1 (strongly disagree) to 5 (strongly agree).

## Results

The participants had mild to moderately severe PD (Table 5). Seven of the participants played games at home, with most of them playing paper based puzzles such as Sudoku or crosswords (Table 6). Three of the participants played the Nintendo Wii^TM^ at home. Most participants were self-motivated to exercise and all stated they exercised at least once a week (Table 7). Walking was the most common type of exercise mentioned. Most people played games and exercised by themselves, although some did involve friends or family.

**Table 5:** Description of participants

**Age (years)**	**Sex**	**Height (m)**	**Mass (kg)**	**Activity balance confidence (%)**	**UPDRS III**	**Hoehn & Yahr stage**
78	M	1.67	77.0	91.9	30	III
61	F	1.55	54.8	97.5	12	I
73	F	1.64	55.0	29.4*	22	II
71	F	1.57	88.2	92.2	9	I
60	F	1.56	69.8	98.6	13	II
54	F	1.78	68.6	99.9	10	I
69	M	1.76	77.8	94.7	23	II
70	F	1.46	60.0	81.9	25	II
78	M	1.73	66.0	94.1	31	II

*Recently diagnosed with hypotension.

**Table 6:** Response to interview questions about games played at home

**Participant**	**What types of games do you play at home?**	**Who do you play with?**	**How often do you play and for how long?**	**Where in your home do you play?**
1	Does not play games	-	-	-
2	Crosswords, polygon, Sudoku, code word	Mainly on my own, although occasionally with partner	1 hr per day	Kitchen, Bedroom or bathroom
3	Crosswords, sometimes pub quizzes	Crosswords alone. Pub quizzes with friends.	4 x 1 hr per week but more when travelling	Living room, Public transport
5	Sudoku, Crosswords, Brainteasers, Solitaire, Scrabble, Nintendo Wii	Alone or with daughters	Paper-based games (30 min-1 hr per day) Nintendo Wii (1 x 20 min per week)	Paper based puzzles (Bedroom) Nintendo Wii (lounge)
6	Sudoku	Myself	Once per week	Kitchen
7	Solitaire, Back gammon, bridge, chess, checkers	With partner or grandchildren	5 x up to 1 hr (plus 10 min at work)	Home office
8	Nintendo Wii, Sudoku, crosswords, pub quiz, checkers, chess, bridge	Alone or occasionally with family	Rarely on the Nintendo Wii Monthly pub quiz Sudoku and crosswords daily	Spare bedroom, Pub
9	Nintendo Wii, various card games, jigsaw puzzles, Sudoku, puzzle books	Friends, mother, daughter, grandchildren	Varies greatly depending who is visiting Monthly friends games night Nintendo Wii 1 x week	Bedroom, lounge, dining room
10	Does not play games	-	-	-

**Table 7:** Response to interview questions about exercise

**Participant**	**What type of exercise do you do/How often?**	**Do you exercise with anyone?**	**Are you self-motivated to exercise?**	**Would you consider the game you just played an exercise game?**	**Did the game support the right type of exercise for you?**
1	Walking (1/2 mile daily)	No. Occasionally with partner	Yes	Not really	It was too easy
2	Horse riding (3 x per week) and stable care (daily). Pilates/physiotherapy exercises (30 min daily). Walk the dog (daily). Gardening (30 min weekly).	Friends, daughter or employee (stable care).	Yes	Yes	It probably was because it made me exercise my arms and shoulders.
3	Walking. Used to enjoy pilates and yoga (3 x weekly) but have stopped 6 weeks ago because of hypotension.	In a group	Was self-motivated before problems with hypotension	Yes	I think this is a good balance exercise but feel it would not suit me now because of my hypotension.
5	Yoga (3 x weekly). Aerobic and strength training once weekly. Walk daily.	No. Occasionally with partner.	Yes, mostly.	Yes. It was also cognitively challenging. It’s not like any other physical exercise. It made me use my mind and body.	Yes. I need to be encouraged to do move more. Slow movements can be off-putting but the movements in the game were right for me and didn’t trigger my tremor.
6	Incidental exercise (works on a farm daily). Otherwise no.	No. Although it my children were younger I would feel more motivated to pay with them.	No	It was more a mind exercise. I thought I was thinking more than moving my body.	No. If I thought my balance was poor and this game would help it, I would definitely play this game at home.
7	Walks 1 mile daily.	No	Yes	No. I thought it was more of a game of coordination, reaction time and balance.	Not for me.
8	Walking 3 x weekly	With my partner.	Yes but not enough to go to the gym.	No. It’s not aerobic enough to be considered an exercise.	Yes. It challenges my balance and coordination.
9	Walk about 6,000 steps daily. Circuit exercise class at the gym once weekly. Nintendo Wii once weekly.	I usually exercise alone but sometimes with others.	Yes	Yes. Quite energetic and I felt I used my arms and legs a lot.	I thought this game challenged my coordination but felt that my balance was not challenged enough.
10	Golf twice weekly.	Yes.	Yes.	An exercise of the mind.	I’m not sure. I found the game challenging.

### Gameplay

**Figure 2:**
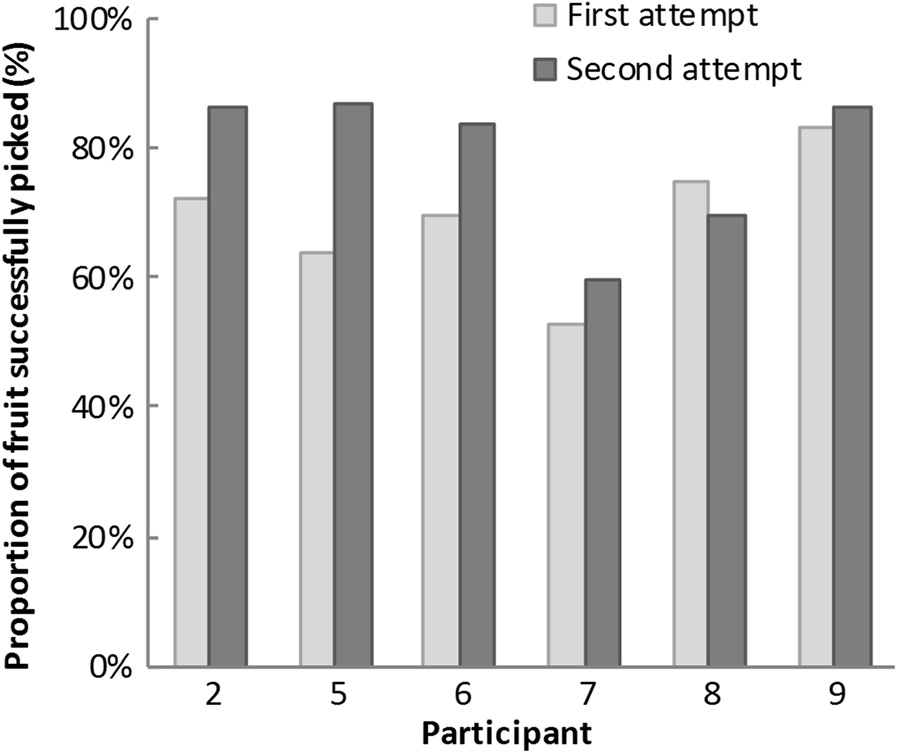
Reaching performance on level 5 (stepping and condition reaching) on the first and second attempts in the 6 participants who progressed through all of the levels.

There were no adverse events although one participant felt dizzy prior to gameplay due to hypotension. Six of the participants progressed through all of the levels of the game, with the remaining three finding the more demanding levels (multi-directional stepping whilst reaching for fruit) too demanding. Whilst playing the game, participants performed a mean of 328 reaching actions (range 167–628 repetitions) and 167 large steps (in multiple directions, range 74–276 repetitions). People performed worse (percentage of fruit successfully gathered per level) on levels where they had to concentrate which hand to pick the fruit with compared to levels where fruit could be picked with either hand (Figure 2).

### Participant feedback

Table 8 summarises participant feedback relating to gameplay. Generally participants reported enjoyment of playing the game and all of the PD participants felt safe whilst playing the game. Seven of the participants stated that they could imagine themselves playing the game at home, although whether or not they would buy the game would depend on the price. Participants said they would enjoy playing the game with others, with competition being an important gameplay factor raised by several people.

**Table 8:** Response to interview questions about gameplay

**Participant**	**What do you think of the game you just played?**	**Could you imagine playing this game at home?**	**Would you want to play the game with anyone else?**	**Did you feel safe playing the game?**	**If the game was available to you to use at home, would you play it?**	**If the game was available to purchase, would you buy it?**
1	Thought it was okay. Didn’t require much thinking. Could be improved by making it faster.	Yes	Not really	Yes	No	No
2	I struggled with depth perception. Couldn’t distinguish the birds from the wasps. When I stepped to one side I thought fruit curved to the side. Difficult to judge the height of the tractor. Some of the play was not realistic (e.g. fruit not on the trees.)	Yes but not often. Possibly when the grandchildren are a bit older or if it was in line with my interests (horses).	Maybe with grandchildren. It would also help if I could play competitively with people.	Yes	I might play it once a week and gradually stop.	Not in its current form. I could not see any real progress once I got the depth and height perception correct.
3	This game would suit me if I could play it with a group.	No	Friends	I felt settled once I started playing*	I doubt it.	No. I have tried DVD exercises which I haven’t found useful.
5	Quite enjoyable. Judging the distance of the fruit and obstacles was difficult, especially when I had to multitask. More practice driving would have been good. Usually I do things slowly but this game encouraged me to move faster.	Yes. It would be good to do things that are specific to the abilities of people with movement disorders.	If playing for fun, yes. If playing competitively, then no.	Yes	Yes. Particularly if I felt it would improve my movement problems.	Yes but it depends on the cost of the whole system.
6	Very good fun. Found it hard to judge the height of fruit.	Yes	It would be good to play this game competitively.	Yes	Yes	I don’t know. Generally I don’t do things like this but I would if I knew that it would improve my movement.
7	I think it has great potential but needs refinement. The graphics look dated and the style childish.	Not in its current form.	I don’t think my grandchildren would be interested.	Yes	Not in its current form.	Not in its current form.
8	Enjoyed the game. There needs to be a greater difference between the birds and wasps. I found the tractor platform was confusing to move. A practice session would be useful.	Yes.	Yes, in a competitive way.	Yes	Yes	It depends on the cost of the game and the associated hardware.
9	It was good and has a lot of potential. I might get bored after a while. The birds and wasps were difficult to differentiate. I found the sensitivity of the driving platform difficult.	Yes.	I could play on my own but it would be fun playing with other people.	Yes	Yes	It would depend on cost.
10	It was interesting. I enjoyed playing the game. I had to keep my mind focussed on what I was doing.	Yes	Yes, with my partner and friends “as long as I can beat them”	Yes	Yes	Yes

*Felt slightly dizzy when first came to the lab (recently diagnosed with hypotension). They rested until they were confident to play the game.

Negative feedback of the gameplay focussed on problems with the visuals, such as the inability to distinguish different objects in the game, such as the birds and the wasps, or the position of the fruit coming towards them. Some people also had some difficulty with stepping to drive the tractor. Interestingly, many perceived the game as more of a cognitive challenge than as a game of balance.

### Flow state scale

The results from the Flow State Scale (Table 5) questionnaire, which quantifies the levels of immersion into the gameplay, showed that the participants experienced states of flow occurring during the game. This was particularly the case with respect to the “concentration” item which showed the highest mean value across participants, indicating that all participants were concentrating a lot on the game. Participants also tended to score highly on the loss of self-consciousness, clear goals and enjoyment items.

## Discussion

### Game design

The first aim of this study was to create an exergame appropriate for people with PD. Exergaming may provide clinicians with an effective therapeutic tool to augment rehabilitation of motor function [38,39] in people with neuropathies such as traumatic brain injury [15,40], cerebral palsy [16,41] and stroke [20,23]. However, it is important that the rehabilitation exergames are designed within the context of the specific rehabilitation needs and capabilities, as well as fitting with the values and lifestyles, of the target population.

The rehabilitation needs and capabilities of people with PD vary greatly between people and over the progression of the disease. One important consideration is that the games for people with PD should not be made too difficult, in terms of their pace or cognitive complexity [25,27]. This concern was also raised in the design workshop and feedback on gameplay in the current study suggested that, although the pace was not a problem, some people found aspects of the game more cognitively than physically challenging. For example, not all of the participants were able to progress through all of the levels of difficulty on the first attempt playing the game, as they found the later levels too complex. In most cases, the aspect of the game participants found most challenging was producing the correct stepping direction in relation to oncoming obstacles under the time pressure of the game. This is understandable considering we sought to expose participants to as much of the gameplay as we could within one session of playing. In a home-based intervention, however, we would suggest people progress slowly through the levels, stopping to practice each component of the game until they felt confident to progress. Alternatively, programming thresholds of performance required before progressing to harder levels may help ensure safety and appropriate practice during home based gameplay.

Another goal of the game was to train postural control under cognitively challenging situations. To this end, we deliberately made the later levels of the game more cognitively demanding. For example, we found participants performed worse on levels where they had to concentrate on which hand to pick the fruit with compared to when they could pick the fruit with either hand. This suggests that the game was able to challenge motor performance under cognitively demanding situations. As discussed previously, however, it is important that progress through the game is paced in such a way that someone playing at home would not progress too fast and risk their safety.

In addition, many of our participants found some visual aspects of the game difficult, whether that be distinguishing the different types of obstacles, the timing of when they had to reach for the approaching fruit or recognising the different positions of the tractor. Impaired visual function has been well described in Parkinson’s disease but its impact on movement is less clear [42-47]. The visual difficulties observed in this study are interesting for two reasons. First, it highlights the need to make the appearance of visual assets in exergames for PD easy to distinguish and their orientation more obvious, as not to distract from the primary challenge of the game which is to improve movement. Second, it is possible that exergames might be useful in identifying and monitoring visuospatial problems in PD.

Overall, the feedback from the pilot testing was positive however we also asked participants to provide constructive feedback to help us identify issues where the game can be improved. We hope that these issues raised may also help other developers produce games appropriate for people with PD. For example, better distinction between game objects, better visual cues as to the timing of approaching objects, a more intuitive driving mechanism and more positive feedback may improve the acceptance of the game. Music and multi-player compatibility may also improve gameplay and enjoyment of the game. In addition, some participants did not feel they would play the game at home if available. Of these, one participant felt the game was too easy, one said the graphics and gameplay would need to be at the standard of commercial games for him to play with his grandchildren, or felt they’d get bored to quickly. A third participant did not respond well to DVD based exercises previously and was apprehensive about investing the time into playing the game if she was not convinced it would improve her performance.

### Safety

All nine of the participants responded that they felt safe whilst playing the game and there were no adverse events during any of the sessions. The physiotherapist attending the sessions, however, noticed that some participants had some difficulties during the stepping tasks. This was most notable when having to step backwards and when under time pressure during more complex levels of gameplay.

### Feasibility

Participants in this study tended to enjoy playing the game and also improved with practice. Results for the Flow State Scale indicated that people with PD were immersed in the gameplay. These findings in the context of the motor learning literature suggest that exergaming could be a potent intervention to improve function in PD. Motor learning studies demonstrate capacity for people with PD to learn a variety of motor tasks ranging from upper limb movement to whole-body functional tasks [6,48,49]. The use of complex task practice rather than repetition of a simple movement has been shown to produce a more pronounced alteration in the neural circuitry suggesting cortical reorganization [5]. A common feature of these studies is the use of enhanced sensory feedback such as auditory pacing cues, visual cues or somatosensory cues to provide augmented feedback about movement performance. Motor learning is enhanced by external cueing [7,8] and action observation [9] whilst clinical studies have shown that externally cued practice over more extended periods (3–6 weeks) leads to significant benefits for gait, balance and transfers [6,10-12] and is more effective than interventions that do not use augmented feedback [6,12,13,50]. These studies represent an exciting and novel area of development that could have potentially important benefits for functional independence. Exergaming represents a way to deliver relevant and motivating training modes that capture all of the above elements of complex skill practice of a wide variety of skills coupled with enhanced sensory feedback are still to be developed.

Home use and tailored training may also facilitate exercise compliance and motivation [51]. Compliance is enhanced as computer games are becoming a normal leisure activity for older adults with the benefits of family and carer participation, and they focus on recreation rather than rehabilitation. Encouragingly, most of the participants in the current study incorporated playing games at home into their leisure time, with three of them already using a Nintendo Wii, and most could imagine themselves using the game at home. Furthermore, exergames are usually designed for home use allowing self-management and monitoring of exercise based therapy. The only home-based exergaming study in PD showed that 18 × 40 minute sessions of playing Nintendo Wii™ fitness based games over 6 weeks was able to elicit improvements in several clinical measures of motor function (such as the 10 m walk and unipedal stance) in a small sample of people with PD [26]. More needs to be known about the compliance and effectiveness of home-based exergaming in PD before widespread adoption as a rehabilitation tool. Despite the promising results in this lab based study, it also remains unclear whether exergaming is safe in a home-based setting for people with PD.

This study was limited in so far as we were not able to establish whether it is as effective at improving postural control, nor how it compares to traditional rehabilitation programs. The small and relatively high functioning sample in our study also limits our ability to generalise to the broader population of people with PD. As older adults also show reduced mobility [52] and dual-task deficit in dynamic postural conditions [53], this game may help improve postural control in older adults without PD as well.

## Conclusion

Exergaming using the Microsoft Xbox Kinect system is safe and feasible for people with PD to use however future home-based intervention studies with a larger sample are required to establish our game’s safety, feasibility and clinical efficacy as a home-based intervention to improve the postural control of people with PD.

## Competing interest

The authors declare that they have no competing interest.

## Authors’ contributions

All authors contributed to the design and implementation of the study. RM, MW and MB designed and ran the design workshop and analysed the data to elicit design requirements. DJ and GS developed the game which was designed based on input from all authors. GB, DM and BG were responsible for data collection and processing of the pilot testing data. PO and LR provided important intellectual input to the manuscript. All the authors contributed to the revision of the manuscript and approved the final version for publication.

## Supplementary Material

Additional file 1 Video of example of gameplay. Click here for file
